# Porcine *E. coli*: Virulence-Associated Genes, Resistance Genes and Adhesion and Probiotic Activity Tested by a New Screening Method

**DOI:** 10.1371/journal.pone.0059242

**Published:** 2013-04-26

**Authors:** Peter Schierack, Stefan Rödiger, Christoph Kuhl, Rico Hiemann, Dirk Roggenbuck, Ganwu Li, Jörg Weinreich, Enrico Berger, Lisa K. Nolan, Bryon Nicholson, Antje Römer, Ulrike Frömmel, Lothar H. Wieler, Christian Schröder

**Affiliations:** 1 Fakultät für Naturwissenschaften, Hochschule Lausitz (FH), Senftenberg, Germany; 2 Department of Veterinary Microbiology and Preventive Medicine, College of Veterinary Medicine, Iowa State University, Ames, Iowa, United States of America; 3 Federal Office of Consumer Protection and Food Safety, Berlin, Germany; 4 Institut für Mikrobiologie und Tierseuchen, Freie Universität Berlin, Berlin, Germany; University of Aberdeen, United Kingdom

## Abstract

We established an automated screening method to characterize adhesion of *Escherichia coli* to intestinal porcine epithelial cells (IPEC-J2) and their probiotic activity against infection by enteropathogenic *E. coli* (EPEC). 104 intestinal *E. coli* isolates from domestic pigs were tested by PCR for the occurrence of virulence-associated genes, genes coding for resistances to antimicrobial agents and metals, and for phylogenetic origin by PCR. Adhesion rates and probiotic activity were examined for correlation with the presence of these genes. Finally, data were compared with those from 93 *E. coli* isolates from wild boars.

Isolates from domestic pigs carried a broad variety of all tested genes and showed great diversity in gene patterns. Adhesions varied with a maximum of 18.3 or 24.2 mean bacteria adherence per epithelial cell after 2 or 6 hours respectively. Most isolates from domestic pigs and wild boars showed low adherence, with no correlation between adhesion/probiotic activity and *E. coli* genes or gene clusters. The gene *sfa/foc*, encoding for a subunit of F1C fimbriae did show a positive correlative association with adherence and probiotic activity; however *E. coli* isolates from wild boars with the *sfa/foc* gene showed less adhesion and probiotic activity than *E. coli* with the *sfa/foc* gene isolated from domestic pigs after 6 hour incubation.

In conclusion, screening porcine *E. coli* for virulence associated genes genes, adhesion to intestinal epithelial cells, and probiotic activity revealed a single important adhesion factor, several probiotic candidates, and showed important differences between *E. coli* of domestic pigs and wild boars.

## Introduction

Intestinal bacterial communities are very complex and individual. Bacteria exist in specific microhabitats and metabolic niches, which are defined by the host (e.g., epithelial cells, mucus, peristaltic activity, pH), nutrition and the microbiota itself. Successful intestinal colonization by a bacterium is associated with many factors, including: adherence to intestinal epithelial cells, induction of lesions, toxin production, and iron acquisition [1]–[3]. Adherence is the first, and most important, component of intestinal colonization. *Escherichia coli,* belonging to the normal microbiota of mammals and birds, express fimbriae and non-fimbrial adhesins to mediate adhesion.

An ingested pathogen must compete with the normal microbiota and can preferentially infect the host if an intestinal niche is unoccupied by another bacteria, or can outcompete an established bacteria in its niche. Thus, it is reasonable that bacteria of the normal microbiota may inhibit infections by intestinal pathogens if they can out-compete the pathogen for a particular niche. Indeed, *E. coli* has been shown to inhibit intestinal infection by certain pathogens [1]–[3]. Such an inhibiting effect is known as a “probiotic” effect.

Though there is no final definition for a “probiotic bacterium”, probiotic bacteria are, in general, living, non-pathogenic microorganisms, which positively affect health and efficiency of human and animal hosts [4], [5]. Probiotics were initially isolated from humans, animals or the environment. For probiotics to be used with confidence, their positive effects must be validated, while their negative, health-concerning effects must be excluded [5]. Probiotics have been shown to influence almost all areas of wellness and health of humans and animals.

One well-studied probiotic is *E. coli* Nissle 1917 (EcN; Mutaflor). Several *in vivo* studies demonstrated its promising probiotic activity in humans and animals [6]–[11]. *in vitro*, EcN has been shown to prevent invasion of host cells by several pathogens, including *Salmonella*, *Yersinia*, *Shigella*, *Legionella*, *Listeria* and adherent-invasive *E. coli* [12]–[14].

In a previous study, we showed that EcN and another F1C fimbriae-positive *E. coli* isolate from a wild boar inhibited *Salmonella* adhesion and invasion. In contrast, F1C fimbriae-negative *E. coli* isolates did not [2]. In this study, the probiotic effects associated with the bacterial adhesion rate and we concluded that adhesion is a prerequisite for probiotic activity. F1C fimbriae have been shown to play an important role in EcN biofilm formation, adherence to intestinal epithelial cells *in vitro,* and intestinal colonization of mice [15], [16].

In the present study, we investigated a large cohort of intestinal *E. coli* isolates from domestic pigs and wild boars for the first time to address the probiotic phenomenon. We searched for genes which supported adhesion of porcine *E. coli* isolates to porcine intestinal epithelial cells and a probiotic activity against an infection by enteropathogenic *E. coli* (EPEC), and verified whether adhesion is a prerequisite for this probiotic activity.

Initially, intestinal *E. coli* isolates from clinically healthy domestic pigs were tested for the occurrence of virulence-associated genes (VAGs) typical for *E. coli* isolated from diarrheic pigs (iVAGs), VAGs typical for extraintestinal pathogenic *E. coli* (eVAGs) and genes coding for resistance against antimicrobial agents (aRGs). Additionally, we tested for genes coding for resistance against metal ions (mRGs), which have not previously been studied in porcine *E. coli*. All *E. coli* isolates were affiliated to phylogenetic groups. In order to cope with the extensive testing for adhesion rates to IPEC-J2 cells and the probiotic effect against EPEC, an automatic analysis of adherent *E. coli* and classification of EPEC microcolonies by modern image analysis on the Aklides® platform was developed. Finally, results from *E. coli* from domestic pigs were compared with results from *E. coli* from wild boars. Data of iVAGs and some eVAGs of *E. coli* from wild boars were from a recent study [17], while data on other eVAGs, aRGs and mRGs, adhesion, and probiotic effects of *E. coli* from wild boars were generated in the present study.

## Materials and Methods

### 1. Bacterial isolates used in this study

Hemolytic *E. coli* isolates have to be excluded from cell culture assays as they destroy cell monolayers due to their hemolytic activity (own unpublished observation). Hemolysis of *E. coli* was tested by transferring isolates on LB agar medium supplemented with 5% sheep blood. Plates were incubated at 37°C and hemolysis was monitored 24 h after incubation. Non-hemolytic *E. coli* isolates from clinically healthy domestic pigs were sampled from feces or intestinal contents of the colon of domestic pigs from 24 pig farms (federal states of Berlin, Brandenburg, Lower Saxony, Saxony, Thuringia and Schleswig-Holstein, Germany). Non-hemolytic *E. coli* isolates from wild boars were sampled from feces or intestinal contents of the colon of wild boars. Samples were taken during several hunts in four different regions of Eastern Germany. These hunts took place in accordance with German hunting rights. Intestinal content or feces of domestic pigs were directly streaked onto CHROMagar orientation plates (Mast Diagnostics, UK) [18]. Some colon samples and swab probes of wild boars were transported on ice and processed as previously described within 24 h [19], [20]. For further confirmation pink colonies from CHROMagar were plated to GASSNER agar. Pink coliform colonies on CHROMagar which were blue/green/yellow coliform on GASSNER agar were assumed to be *E. coli* as demonstrated in several other studies [20]–[22]. One single pink colony was subcultured twice on CHROMagar orientation plates and stored in 10% glycerol at −80°C until further processing. Finally, after PFGE assignment all isolates were verified as *E. coli* using Kligler agar, LIM agar, urea agar and citrate agar [23] or using an *E. coli*-specific PCR for the detection of the *uspA* gene [24].

To exclude identical *E. coli* isolates from further examinations we assigned individual *E. coli* isolates to pulsed field gel electrophoresis (PFGE) types by pulsed-field gel electrophoresis. A PFGE type is an isolate or group of isolates that can be distinguished from other isolates of the same genus and species by macrorestriction analysis [25]. All isolates of domestic pigs were assigned to PFGE types in our actual study using macrorestriction analysis according to a previous published protocol [20]. All isolates from wild boars were assigned to PFGE types during a recent study [17]. Bacteria were embedded in agarose brackets and bacterial DNA was digested with XbaI. DNA fragment patterns were examined by ethidium bromide staining. If XbaI failed to digest DNA 100 µM thiourea were added to the running buffer to avoid DNA degradation by Tris radicals [26]. Finally, 104 PFGE types of domestic pig *E. coli* and 93 PFGE types of wild boar *E. coli* were detectable and included in further analysis.

*E. coli* Nissle 1917 DSM6601 (O6:H1:K5, EcN) was kindly provided by G. Breves (Hannover, Germany). EPEC 6105 (O26:K60) was isolated from a diarrheic pig and kindly provided by R. Bauerfeind (Giessen, Germany). This study was approved by the Ministry of Environment, Health and Consumer Protection of the Federal State of Brandenburg, Germany (V3-2347-8-39-1-2011).

### 2. PCR tests

All *E. coli* isolates from domestic pigs were tested for iVAGs, eVAGs, phylogenetic affiliation, aRGs and mRGs. With some exceptions, *E. coli* isolates from wild boars had been already tested for these iVAGs, eVAGs and phylogenetic assignment during a recent study but with similar PCR conditions [17]. In the present study these isolates have been additionally tested for the eVAGs *aatA*, *fimH*, *H7*, *Yqi*, *iutA*, *astA* and for all aRGs and mRGs. The presence of iVAGs, eVAGs, aRGs and mRGs was tested by PCR using bacterial lysates as previously described [27]–[34]. All iVAGs, eVAGs, aRGs and mRGs are listed in Tables 1 and 2.

**Table 1:** Virulence-associated genes typical for *E. coli* isolated from diarrheic pigs (iVAGs) and isolated from extraintestinal disease (eVAGs) and phylogenetic origin of 104 porcine non-hemolytic *E. coli* isolates.

gene(s) or operon	description	prevalence in *E. coli* (n = 104) from healthy pigs of this study in%	prevalence in *E. coli* (n = 93) from wild boars of the study Römer et al. [17] and of our actual study (#) in%
iVAGs 1			
*est-1a*	Heat stabile enterotoxin 1	0	3.2
*est-2*	Heat stabile enterotoxin 2	2.9	3.2
*fedA*	Subunit of F18 fimbriae	0	1.1
*stx2e*	Shigatoxin 2e	1.9	5.4
*fanA*	Subunit of F5 fimbriae	0	0
*fimF41a*	Subunit of F41 fimbriae	0	0
*fasA*	Subunit of F6 fimbriae	1.0	0
*eltB-Ip*	Heat labile enterotoxin 1	0	0
*faeG*	Subunit of F4 fimbriae	0	0
eVAGs 2			
Adhesins			
*aatA*	APEC autotransporter	16.3*	1.1 #*
*afa/draB*	Afimbrial/Dr antigen-specific adhesin	1.0	0
*csgA*	Curli fibre-encoding gene	78.8	87.1
*fimC*	Type 1 fimbriae (D-mannose specific adhesin)	91.3*	100*
*fimH*	Type 1 fimbriae (D-mannose specific adhesin)	96.1*	83.9 #*
*hra*	Heat-resistant agglutinin	13.5*	23.7*
*H7*	H7 flagellar antigen	15.4	11.8 #
*iha*	Iron-regulated-gene-homologue adhesin	0*	5.4*
*mat*	Meningitis-associated and temperature-regulated fimbriae	81.7*	48.4*
*papAH*	Pilus associated with pyelonephritis	1.9	0
*papC*	Pilus associated with pyelonephritis	9.6*	0*
*papEF*	Pilus associated with pyelonephritis	4.8	0
*papGII*	Pilus associated with pyelonephritis	5.8	0
*papGIII*	Pilus associated with pyelonephritis	1.0	0
*papGII/III*	Pilus associated with pyelonephritis	0	0
*sfa/focCD*	S fimbriae (sialic acid-specific) and F1C fimbriae	4.8	5.4
*tsh* 1	Temperature sensitive hemagglutinin	16.3*	4.3*
*yqi*	APEC fimbrial adhesin	27.9	30.1 #
Iron acquisition			
*chuA*	Heme receptor gene (*E. coli* haem utilization)	19.2*	39.8*
*feoB*	Major bacterial ferrous iron transporter, iron(II) transport system	98.1*	73.1 #*
*fyuA*	Ferric *Yersinia* uptake (yersiniabactin receptor)	26.9	30.0
*ireA*	Iron-responsive element (putative catecholate siderophore receptor)	9.6	3.2
*iroN* 1	Catecholate siderophore (salmochelin) receptor	21.2*	10.8*
*irp2*	Iron repressible protein (yersiniabactin synthesis)	22.1	19.4
*iucD* 1	Aerobactin synthesis	26.0*	1.1*
*sitD* chr.	*Salmonella* iron transport system gene	1.0*	11.8*
*sitD* ep.^1^	*Salmonella* iron transport system gene	14.4*	5.4*
*iutA*	Aerobactin receptor	36.5*	5.4 #*
Protectins/Serum resistance			
*cvi/cva* 1	Structural genes of colicin V operon (Microcin ColV)	19.2*	6.5*
*neuC*	K1 capsular polysaccharide	2.9	0
*kpsMT* II	Group II capsule antigens	21.2	24.7
*ompA*	Outer membrane protein	94.2*	100*
*traT* 1	Transfer Protein	51.9	45.2
Toxins			
*astA*	EAST1 (heat stable cytotoxin associated with enteroaggregative *E. coli*)	43.3	39.2 #
*sat*	Secreted autotransporter toxin	1.0	3.2
*hlyA*	Haemolysin A	3.8	0
*cnf1/2*	Cytotoxic necrotising factor 1/2	6.7	10.8
Invasins			
*gimB*	Genetic island associated with neonatal meningitis	0	0
*ibeA*	Invasion of brain endothelium	3.8*	16.1*
*tia*	Toxigenic invasion locus in ETEC isolates	3.8	5.4
Miscellaneous			
*pic*	Serine protease autotransporter	4.8	6.5
*malX*	Pathogenicity-associated island marker CFT073	7.7	16.1
*ECoR group*			
A		63.5*	29.0*
B1		17.3*	32.3*
B2		4.8*	21.5*
D		14.4	17.2

Comparison to *E. coli* from wild boars of an already published study.

* differences between both groups statistically significant with p<0.05

n.t. = not tested

1 iVAGs were included since it was shown that an iVAG can affect colonization [58].

2 eVAGs were included since it was shown that eVAGs correlated with successful intestinal *E. coli* colonization in pigs [29].

**Table 2:** Resistance against antimicrobial substances (aRGs) and against metal ions (mRGs) of 197 porcine non-hemolytic *E. coli* isolates.

gene(s) or operon	description	prevalence in *E. coli* (n = 104) from healthy pigs of this study in%	prevalence in *E. coli* (n = 93) from wild boars this study in%
aRG 1			
*aadA*	streptomycin-spectinomycin resistance	1.3	0
*bla_TEM_*	ampicillin resistance	17.9*	1.1*
*dfr1*	trimethoprim resistance	7.7*	0*
*qacEdeltaI*	quaternary ammonium compound resistance	6.4	2.2
*qnr*	fluoroquinolone resistance	0*	4.3*
*tetA*	tetracycline resistance	5.1*	1.1*
*tetB*	tetracycline resistance	12.8*	0*
*sul1*	sulfonamide resistance	2.6*	0*
mRG 2			
*arsC*	arsenic resistance	94.2*	100*
*merA*	mercury resistance	2.9	2.2
*pcoA*	copper resistance	12.5*	0*
*pcoD*	copper resistance	9.6*	0*
*pcoE*	copper resistance	12.5*	0*
*silE*	silver resistance	13.5*	2.2*
*silP*	silver resistance	8.7	0*
*terD*	tellurite resistance	4.8	0*
*terF*	tellurite resistance	1.9	0
*terX*	tellurite resistance	1.9	0
*terY3*	tellurite resistance	1.9	0

1 aRGs were included since aRGs affect colonization which is known as “the cost of antimicrobial resistance” [59].

2 mRGs were included since there were no data on occurrence of such genes in *E. coli*. We supposed that mRGs affect colonization similar to aRGs.

*E. coli* isolates were classified according to the ECoR (*E. coli* Reference Collection) system [35] by use of the rapid phylogenetic grouping PCR technique described by Clermont et al. (2000) [36]. According to this method, isolates were assigned to one of four groups (A, B1, B2 or D) based on their possession of two genes (*chuA* and *yjaA*) and the DNA fragment TspE4.C2.

### 3. In vitro assays

#### 3.1. IPEC-J2 cell culture conditions

The porcine intestinal epithelial cell line IPEC-J2 [37] was grown in Dulbecco's modified Eagle Medium (DMEM) HAM`S/F-12 (1∶1) (Biochrom, Germany), supplemented with 5% fetal calf serum, and maintained in an atmosphere of 5% CO_2_ at 37°C. Cells reached confluence after 3–4 days and were used within 8 days from seeding.

#### 3.2. *E. coli* adhesion assays

Adhesion assays were performed essentially as previously described [2]. *E. coli* were grown overnight in LB medium to an optical density at 600 nm (OD_600_) of 0.8–1.2. IPEC-J2 cells were inoculated with *E. coli* with a multiplicity of infection (MOI) of 100∶1 *E. coli* to host cells in culture wells of 96-well plates (Greiner bio-one, Germany) using a conversion of approximately 3×10^8^ bacteria/ml/OD_600_. Confluent monolayers of IPEC-J2 cells were incubated with the respective *E. coli* isolate for 2 or 6 hours at 37°C. 2 or 6 hours were included to understand differences of adhesion dynamics between isolates. Cells were washed three times with 1xPBS to remove non-adherent *E. coli*. Cells with adherent bacteria were fixed with paraformaldehyde in 1xPBS (4%) and kept at 4°C until further usage. Before analysis paraformaldehyde was removed by washing plates three times with 1xPBS. The last washing step also included a 15-minute incubation step with propidium iodide (10 µg/ml in 1xPBS). After the last washing step, plates were dried at room temperature. Finally, plates were analysed by the Aklides® system (Medipan, Dahlewitz/Berlin, Germany, [38]). All assays were done in duplicate wells and were repeated at least once.

#### 3.3. EPEC infection assays

EPEC 6105 was grown in LB medium to an OD_600_ of approximately 1. Confluent monolayers were infected with a MOI of 100∶1 EPEC to host cells using a conversion of approximately 3×10^8^ bacteria/ml/OD_600_. Confluent monolayers were infected for three hours with EPEC 6105 after a 2 h *E. coli* pre-incubation period (see 2.4.2. *E. coli* adhesion assays) with subsequent washing (thrice with 1xPBS). After 3 h non-adherent bacteria were removed by three washes with 1xPBS and incubation was continued for further 3 h. Cells were again washed thrice with 1xPBS. Cells with adherent bacteria were fixed with paraformaldehyde in 1xPBS (4%) and kept at 4°C until further usage. Before analysis paraformaldehyde was removed by washing plates three times with 1xPBS. After blocking with blocking buffer (0.5% albumin fraction V in 1× PBS) for 5 min EPEC were stained with primary antibodies against O26:K60 (dilution 1∶50, Sifin, Germany) in blocking buffer for 30 min at room temperature. Cells and bacteria were washed thrice with blocking buffer. EPEC were stained with secondary antibodies (anti-rabbit antibodies, IgG, FITC-labelled, dilution 1∶200) for 30 min at room temperature in dark. Cells and bacteria were washed thrice with blocking buffer, incubated with DAPI for 30 sec and again washed thrice with 1× PBS. Finally, plates were analysed by the Aklides® system. All assays were done in duplicate wells and were repeated at least once. *E. coli* strain Nissle 1917 was included as positive control in the EPEC infection (probiotic) assays [2], [39].

### 4. Basic principles of the new Aklides software module

*E. coli* isolates were stained with propidium iodide, EPEC with fluorescence-labelled antibodies and nuclei of IPEC-J2 cells with propidium iodide or DAPI. The Aklides® system automatically captured images of cell monolayers with adherent fluorescent bacteria employing basic software algorithms successfully implemented for pattern recognition of cell-based indirect immunofluorescence for autoantibody detection in routine diagnostics of systemic rheumatic diseases [40]–[43]. Successful automation of double strand DNA break assessment by indirect immunofluorescence could be demonstrated with the same system [44].

Briefly, analysis of stained bacteria included focusing on each well, acquisition of multiple images in one wavelength (490 nm) and counting and characterization of fluorescent objects. From each well of a 96-well cell culture plate 5 images were taken with a 40× objective in a spiral manner beginning from the centre of the well. For each position, focusing algorithm captured step by step images in different z-positions inside a certain range and detected images with highest focus level. Subsequently, different image processing algorithms were applied to select images. Background and object separation was done by histogram based thresholding using modus as expected background intensity followed by segmentation of objects with an 8 pixel neighbourhood. Thereafter oversize objects, typically staining artefacts, were excluded by overwriting them with background intensity level.

In each picture numbers of cell nuclei and numbers of bacteria were counted. Cell nuclei and bacteria were defined by their minimal and maximal a) size ( µm), b) shape (axis ratio), and c) fluorescence intensity. Counting numbers of cell nuclei was beneficial for quality control of intact cell monolayers. An EPEC microcolony was defined by existence of bacteria sticking to each other. EPEC microcolonies were grouped by their size: 1) single bacteria (no microcolony), 2) small microcolonies (2–10 bacteria), 3) medium-sized microcolonies (11–20 bacteria) and 4) large microcolonies (more than 20 bacteria).

### 5. Statistical analysis

All statistical tests were performed with RKWard, version 0.57 using dedicated R packages and custom made scripts or RKWard plugins [45]. Robust linear regression was done with an MM-type regression estimator. The Pearson's product moment correlation coefficient was used to estimate the association between samples. The Mann-Whitney test was used to assess differences between two groups. Probability values of less than 0.05 were considered statistically significant. The dendrogram was used to estimate the genetic relationships among isolates using a manhattan distance measure and a single agglomeration method.

## Results

### 1. Phylogenetic affiliation; virulence-associated genes and genes for resistance against antimicrobial agents and metal ions

Initially, from clinically healthy domestic pigs 104 non-hemolytic putative *E. coli* isolates each belonging to a unique PFGE type were tested for the occurrence of the *E. coli*-specific *uspA* gene [24]. Three isolates, which had typical morphological and biochemical characteristics of *E. coli*, did not carry this gene. These strains were additionally typed by Maldi-TOF, were all identified as *E. coli* (data not shown) and were, thus, included in further analysis. Phylogenetic affiliation and presence of iVAGs and eVAGs in these 104 isolates are listed in Table 1. Most isolates belonged to ECoR group A (63.5% of isolates) followed by groups B1 (17.3%), D (14.4%) and B2 (4.8%). iVAGs were only sporadically detected with genes present *est-2* (2.9%), *stx2e* (1.9%) and *fasA* (1.0%). Isolates carried from 3 to 22 eVAGs (median 8.5 eVAGs) with *iha* and *gimB* absent in all isolates and *feoB* (97.1%), *fimH* (96.2%), *ompA* (94.2%), *fimC* (91.3%), *mat* (81.7%) and *csgA* (78.8%) most present in isolates. All 104 isolates were additionally tested for aRGs and mRGs and results are shown in Table 2. Isolates carried from 0 to 5 aRGs (median 0 aRGs) with *qnr* absent in all isolates and *bla_TEM_* (15.4%), *tetB* (14.4%) and *tetA* (10.6%) most present in isolates. Isolates carried from 0 to 10 mRGs (median 1 mRGs) with no absent mRG and *arsC* (94.2%) and *silE* (13.5%) most present in isolates.

Comparisons of iVAGs, eVAGs, aRGs and mRGs between *E. coli* from clinically healthy domestic pigs and wild boars are included in Table 1 and 2. Wild boar isolates carried from 4 to 20 eVAGs (median 8 eVAGs) with *fimC* (100%), *ompA* (100%), *csgA* (87.1%), *fimH* (83.9%) and *feoB* (73.1%) most present. Isolates carried from 0 to 1 aRGs (median 0 aRGs) with *aadA, dfr1, tetB* and *sul1* absent in all isolates. Other aRGs occurred only very sporadically. Isolates carried from 1 to 2 mRGs (median 1 mRGs). All mRGs were absent with the exception of *arsC* (100%), *merA* (2.2%) and *silE* (2.2%). We additionally tested these wild boar isolates for the *E. coli* typical *uspA* gene. Twelve isolates from wild boars of this recent study [17], which had typical morphological and biochemical characteristics of *E. coli*, did not carry *uspA*. However, these twelve isolates were typed by Maldi-TOF as *E. coli* (data not shown) and were included in our adhesion and probiotic assays.

### 2. Adhesion of *E. coli* isolates to IPEC-J2

*E. coli* isolates were incubated with IPEC-J2 over 2 and 6 h. Non-adherent *E. coli* were removed, and after staining with propidium iodide, absolute numbers of adherent bacteria were counted using the Aklides® system.

#### 2 h incubation period

**Figure 1:**
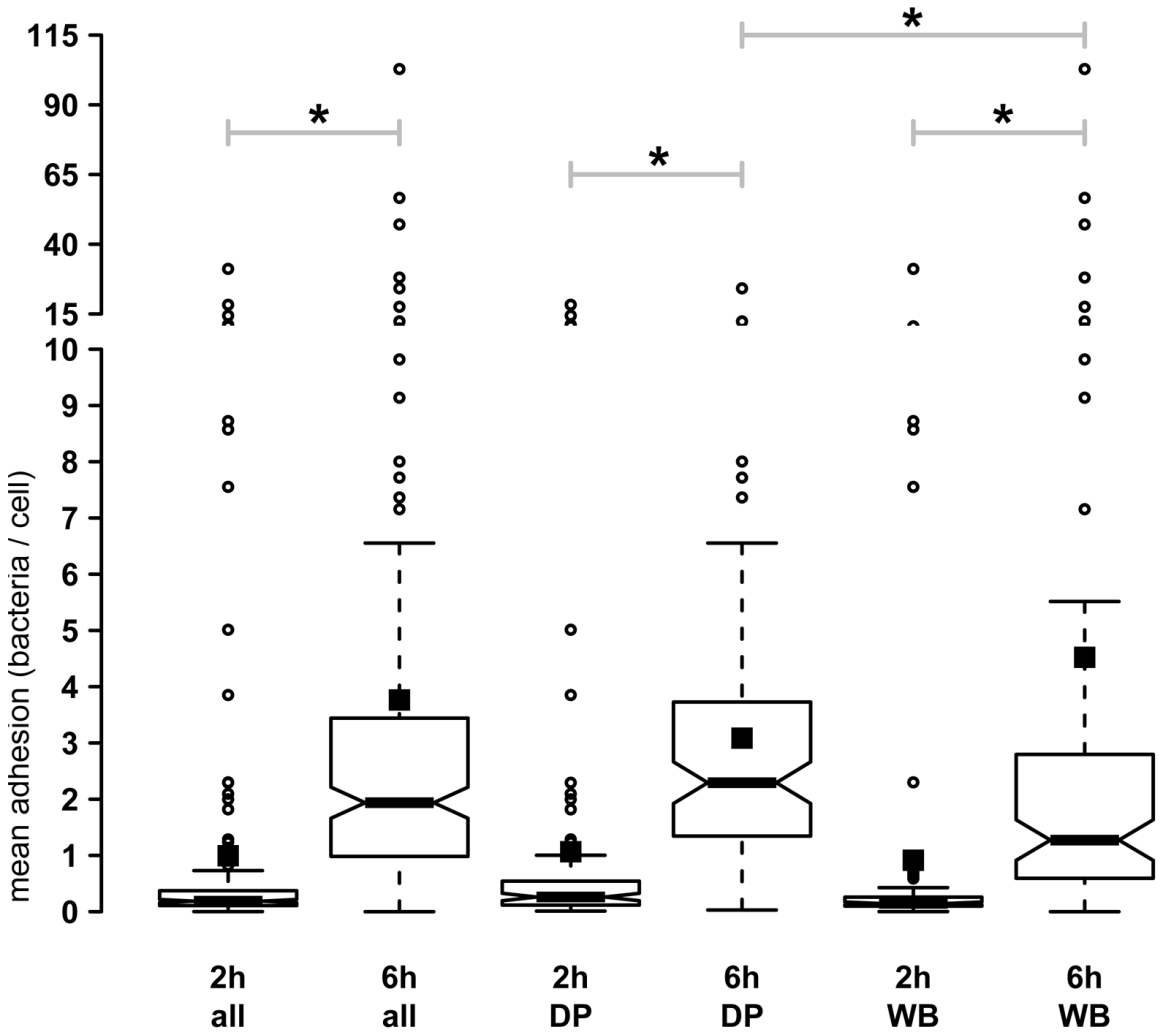
Adhesion rates of *E.* *coli* to IPEC-J2. IPEC-J2 cells were incubated with *E. coli* from domestic pigs (DP, n = 104) or wild boars (WB, n = 93) over 2 h or 6 h. Adhesion was quantified after removing non-adherent bacteria by washing. There were significant differences in adhesion following 2 h and 6 h incubation periods. Additionally, there was a significant difference in adhesion between *E. coli* from domestic pigs and wild boar after 6 h incubation. All: isolates of domestic pigs and wild boars together, *: p<0.05 between the groups connected by the beam. Solid squares represent the sample mean.

Adhesion of *E. coli* from domestic pigs strongly varied between isolates with 0 to 18.3 bacteria per IPEC-J2 cell (Figure 1). Most isolates (n = 90) had low adhesion rates with less than 1 bacterium per epithelial cell but 6 isolates adhered with more than 5 bacteria per epithelial cell (Table 3). Adhesion of *E. coli* from wild boars also strongly varied between isolates with 0 to 10.6 bacteria per IPEC-J2 cell (Figure 1). Most isolates (n = 88) had low adhesion rates with less than 1 bacterium per epithelial cell but 4 isolates adhered with more than 5 bacteria per epithelial cell (Table 3). There were no statistical differences between adhesion rates of *E. coli* from domestic pigs and wild boars after the 2 h incubation period.

**Table 3:** Adhesion of *E. coli* to IPEC-J2.

	<0.1	0.1<x<1.0	1.0<x<5.0	>5.0
	bacteria	bacteria	bacteria	bacteria
	per cell	per cell	per cell	per cell
domestic pig *E. coli* (n = 104)				
after 2 h incubation	20*	70	8	6
after 6 h incubation	1	14	72	17
wild boar *E. coli* (n = 93)				
2 h	25	63	1	4
6 h	8	28	47	9
all *E. coli* (n = 197)				
2 h	45	133	9	10
6 h	9	42	119	26

Comparison between 2 h and 6 h incubation; classification according to adhesion rate (number of all EPEC bacteria present on IPEC-J2 cells)

* number of strains which adhered with less than 0.1 bacteria per one epithelial cell.

#### 6 h incubation period

Adhesion of *E. coli* from domestic pigs strongly varied between isolates from 0 to 24.2 bacteria per IPEC-J2 cell (Figure 1). Several isolates (n = 15) had a low adhesion rate with less than 1 bacterium per epithelial cell. Seventeen isolates adhered with more than 5 bacteria per epithelial cell (Table 3). Adhesion of *E. coli* from wild boars strongly varied between isolates from 0 to 56.7 bacteria per IPEC-J2 cell (Figure 1). Several isolates (n = 36) had a low adhesion rate with less than 1 bacterium per epithelial cell. Nine isolates adhered with more than 5 bacteria per epithelial cell (Table 3). Adhesion of wild boar *E. coli* was lower than adhesion of *E. coli* from domestic pigs (p<0.05, Figure 1).

### Comparison between the 2 h and 6 h incubation period

After 6 h incubation, the adhesion rates of *E. coli* from domestic pigs and wild boars were statistically higher than after 2 h incubation (p<0.05, Figure 1). Adhesion rates of three *E. coli* isolates from domestic pigs increased up to twofold, of 21 isolates up to fivefold, of 27 isolates up to tenfold and of 43 isolates more than tenfold higher than what was seen after 2 h incubation. However, nine isolates had a decreased adhesion rate after 6 h, as compared to 2 h. Adhesion rates of 6 *E. coli* isolates of wild boars increased up to twofold, of 17 isolates up to fivefold, of 26 isolates up to tenfold and of 34 isolates more than tenfold of the 2 h incubation time. However, adhesion rates of ten wild boar *E. coli* isolates decreased from 2 h to 6 h incubation time.

The most remarkable increase seen in adhesion of an *E. coli* isolate (from a wild boar) was the increase from 0.0079 at 2 h incubation to 9.82 bacteria per IPEC-J2 cell at 6 h incubation. The most remarkable decrease (one *E. coli* isolate from a domestic pig) was the decrease from 10.7 (2 h incubation) to 1.2 (6 h incubation) bacteria per IPEC-J2 cell.

### 3. Associations between virulence-associated genes, genes for resistance against antimicrobial agents and metal ions and phylogenetic affiliation and adhesion

**Figure 2:**
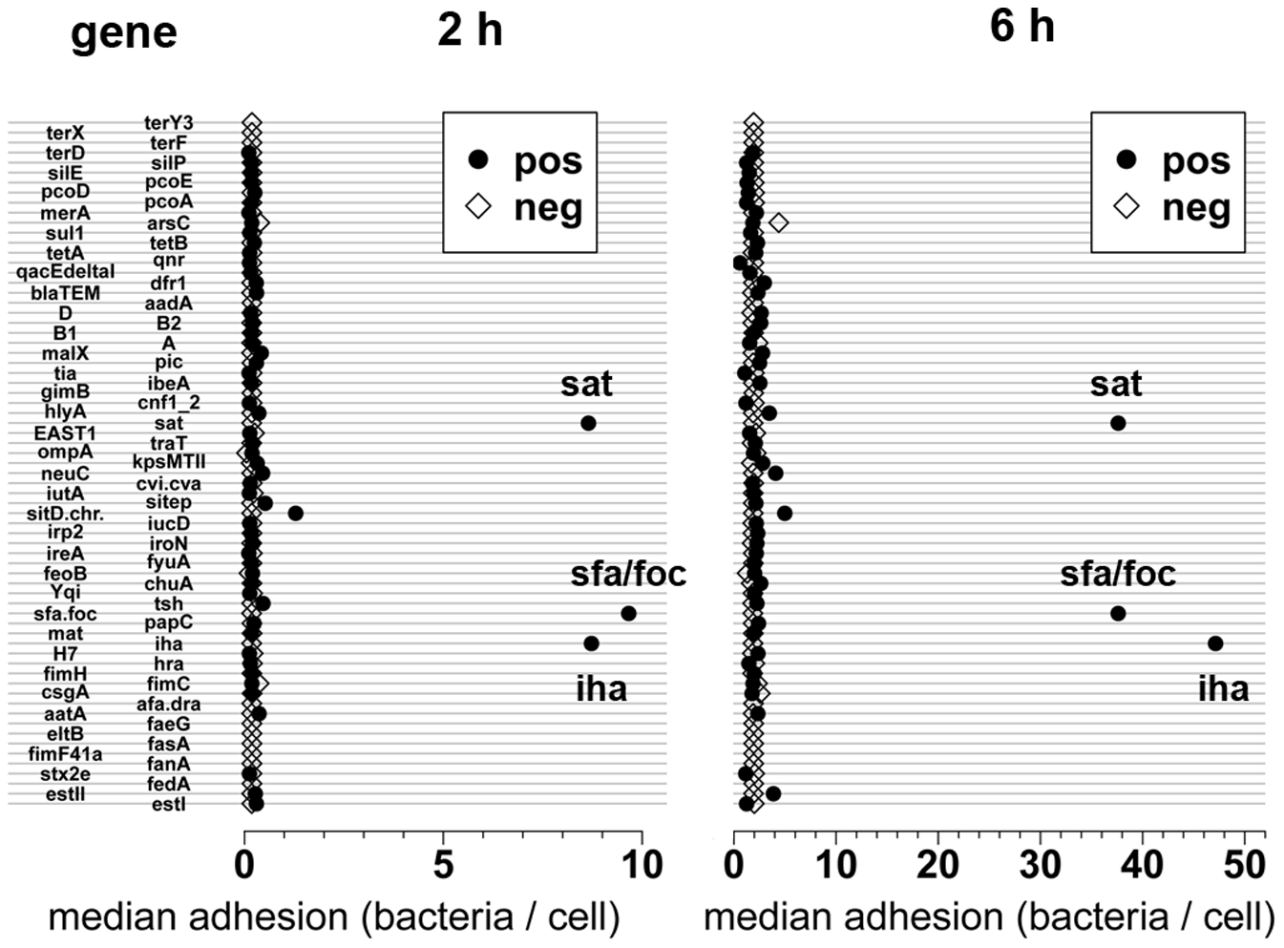
Associations between adhesion rates and a gene. IPEC-J2 cells were incubated with *E. coli* over 2 h or 6 h. Adhesion was quantified after removing non-adherent bacteria by washing. All *E. coli* isolates were grouped being positive (pos) or negative (neg) for a gene. Median of adhesion rates of one group is represented by one dot. There were three significant associations between adhesion and a specific gene (*sfa*/*foc*, *iha*, *sat*).

We had already shown that F1C fimbriae can support adhesion of *E. coli* to IPEC-J2 cells [2]. Here, one isolate from domestic pigs and five from wild boars carried *sfa/foc*, encoding a subunit of F1C fimbriae. Indeed, all these isolates had high adhesion rates after the 2 h (7.6–31.2 bacteria per epithelial cell) and 6 h (28.1–56.7 bacteria per epithelial cell) incubation times. Other genes significantly associated with high adhesion were *sat* and *iha* (Figure 2). However, both genes were also associated with the presence of *sfa/foc*. Three of four strains, which were *sat*-positive, were also *sfa*/*foc*-positive and highly adherent. The one *sat*-positive strain, which was *sfa/foc*-negative, was lowly adherent. Additionally, we tested 7 other *sat*-positive/*sfa/foc*-negative isolates from our strain collection which included isolates from different animal and human origin, but adhesion of five of these strains to IPEC-J2 were low (data not shown). We concluded that *sat* was not a dominant adhesin. All isolates positive for *iha* were also positive for *sfa/foc*. Data about *iha* occurrence in bacteria of our strain collection is not available yet. The role of *iha* in adhesion should be clarified in future studies with bacteria, which are *iha*-positive but *sfa*/*foc*-negative.

Other significant associations between adhesion rates and the occurrence of single genes were not detected. However, several *sfa/foc*-negative isolates also had high adhesion rates after 2 h and/or 6 h incubation times (Table 4), which indicates the presence on these isolates of other strong adhesins.

**Table 4:** Examples of highly adherent *E. coli* isolates without the *sfa/foc* gene.

Strain	adhesion after 2 h (bacteria/cell)	adhesion after 6 h (bacteria/cell)
4308	10.8*	3.0
4318	18.3	24.2
4322	10.7	1.2
4323	11.2	12.4

* mean from two independent experiments which were additionally done after screening and which verified high adhesion.

We also tested whether the increase of adhesion between the 2 h and 6 h incubation times was associated with a specific gene, which would suggest that expression of genes involved in adherence would change over time and/or in the presence of epithelial cells. However, we did not find such an association.

**Figure 3:**
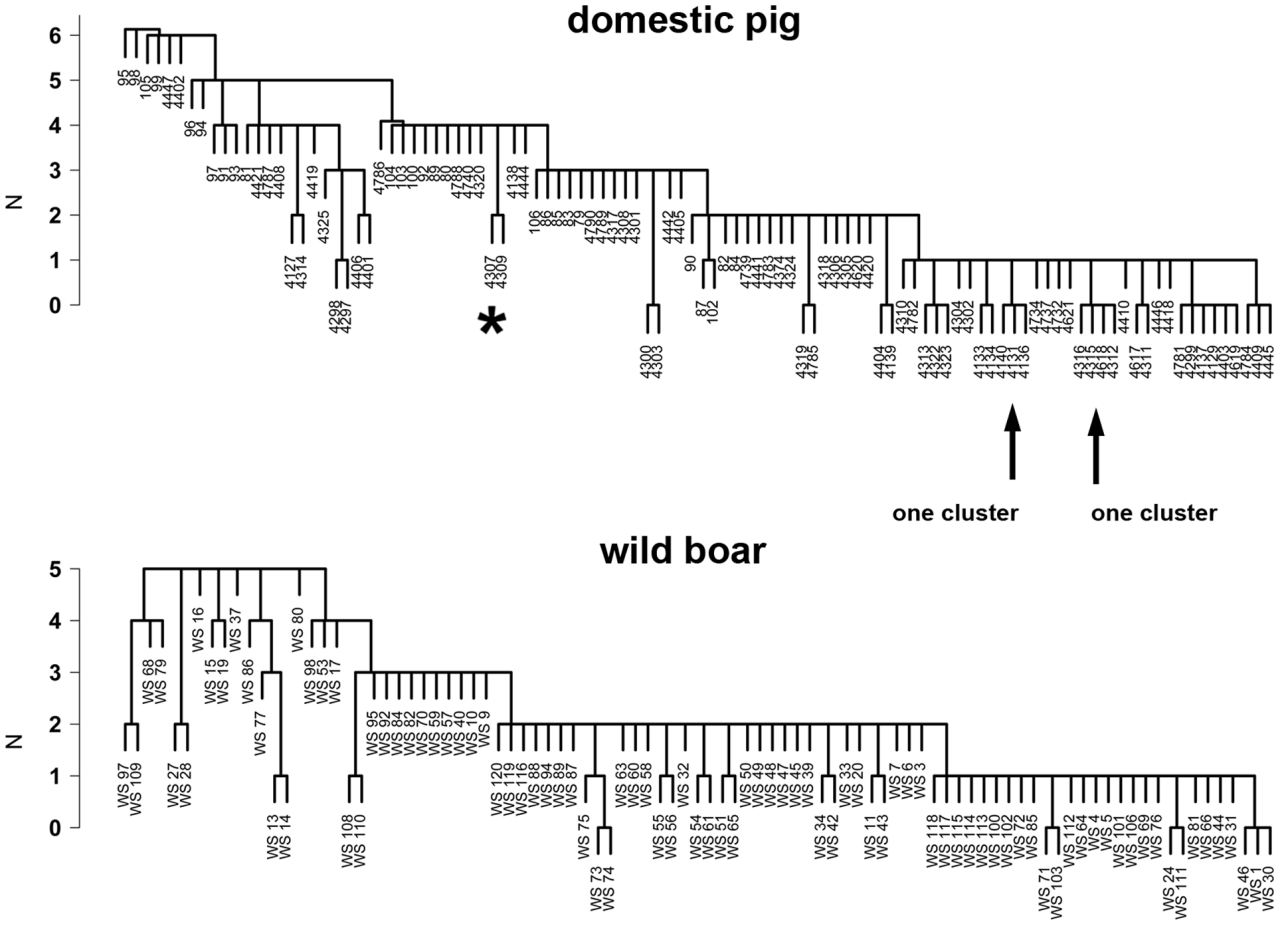
Cluster analysis with virulence-associated genes (VAGs). Isolates from domestic pigs (n = 104) and from wild boars (n = 93) were clustered according to 9 VAGs typical for *E. coli* isolated from diarrheic pigs (iVAGs) and 37 VAGs typical for *E. coli* isolated from extraintestinal disease (eVAGs). Using the manhattan distance measure the distance between strains was computed including a hierarchical cluster analysis using a single linkage method (“friends of friends”) clustering strategy. The minimal difference in the number (N) of genes between clusters of strains to other clusters is described. All isolates of one cluster have identical VAG pattern. Distance of 1 (δN = 1) means that two clusters differed at least in one gene. Clusters which included at least four isolates, which were not different in more than 2 genes, were used to associate virulence gene profiles with adhesion rates or a probiotic effect. There were no associations between a specific cluster and adhesion or a probiotic effect. *: Clustered isolates are identified by strain numbers.

Additionally, we clustered isolates based on eVAGs, aRGs and mRGs (Figure 3) and tested these clusters for association to adhesion after 2 h or 6 h incubation or increase in adhesion. No associations between a specific cluster (gene profile) and adhesion or increase of adhesion were found.

### 4. Effects of *E. coli* isolates on EPEC infection

**Figure 4:**
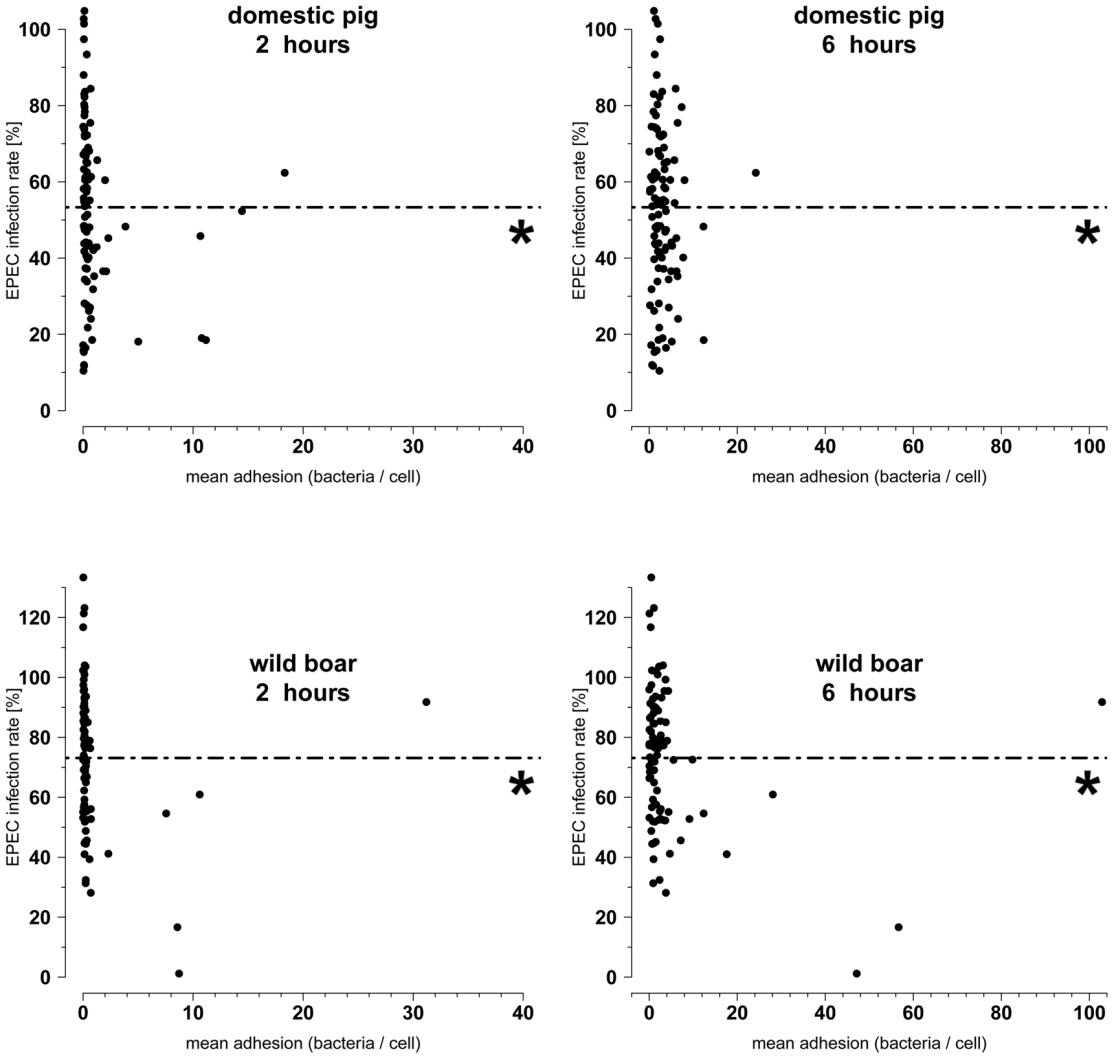
Association of adhesion and a probiotic effect. X-axis: IPEC-J2 cells were incubated with *E. coli* over 2 h or 6 h. Adhesion was quantified after removing non-adherent bacteria by washing. Y-axis: EPEC infection rates were determined in an EPEC inhibition assay: *E. coli* were incubated over 2 h with IPEC-J2 cells. Non-adherent bacteria were removed by washing. Subsequently, IPEC-J2 cells were incubated with EPEC. After 6 h non-adherent bacteria were removed by washing. The EPEC infection rate was calculated in relation to EPEC mono-infection (no domestic pig or wild boar *E. coli* pre-incubation = 100%). Conclusively, a number below 100% indicates a probiotic effect = reduction of EPEC. Isolates from domestic pigs had a higher probiotic effect ( = higher reduction of the EPEC infection rate (p<0.05)). There were no significant associations between the adhesion capabilities of strains and their probiotic effects. *: Mean of EPEC infection rate.

All *E. coli* isolates were tested for their probiotic effect against EPEC. Isolates were pre-incubated over 2 h prior to EPEC infection. Adherent EPEC bacteria were fluorescence-labeled and quantified with the Aklides® system. With the exception of three isolates, all *E. coli* isolated from domestic pigs inhibited the EPEC infection rate (number of EPEC present on IPEC-J2 cells) with 72 isolates inhibiting EPEC infection by more than 50% and two isolates inhibiting EPEC infection by more than 90% (Figure 4). In general, the probiotic effect of one isolate was independent of that isolate's adhesion rate to IPEC-J2 cells (Figure 4) and gene profile (data not shown).

With the exception of eight isolates, all other *E. coli* isolated from wild boars inhibited EPEC infection with 28 isolates inhibiting EPEC infection by more than 50% and one isolate inhibiting EPEC infection by more than 90% (Figure 4). In general, the probiotic effect of an isolate was independent from its adhesion rate to IPEC-J2 cells (Figure 4) and gene profile (data not shown). *E. coli* isolated from domestic pigs inhibited EPEC infection significantly more than the *E. coli* isolated from wild boar (p<0.05, Figure 4).

**Figure 5:**
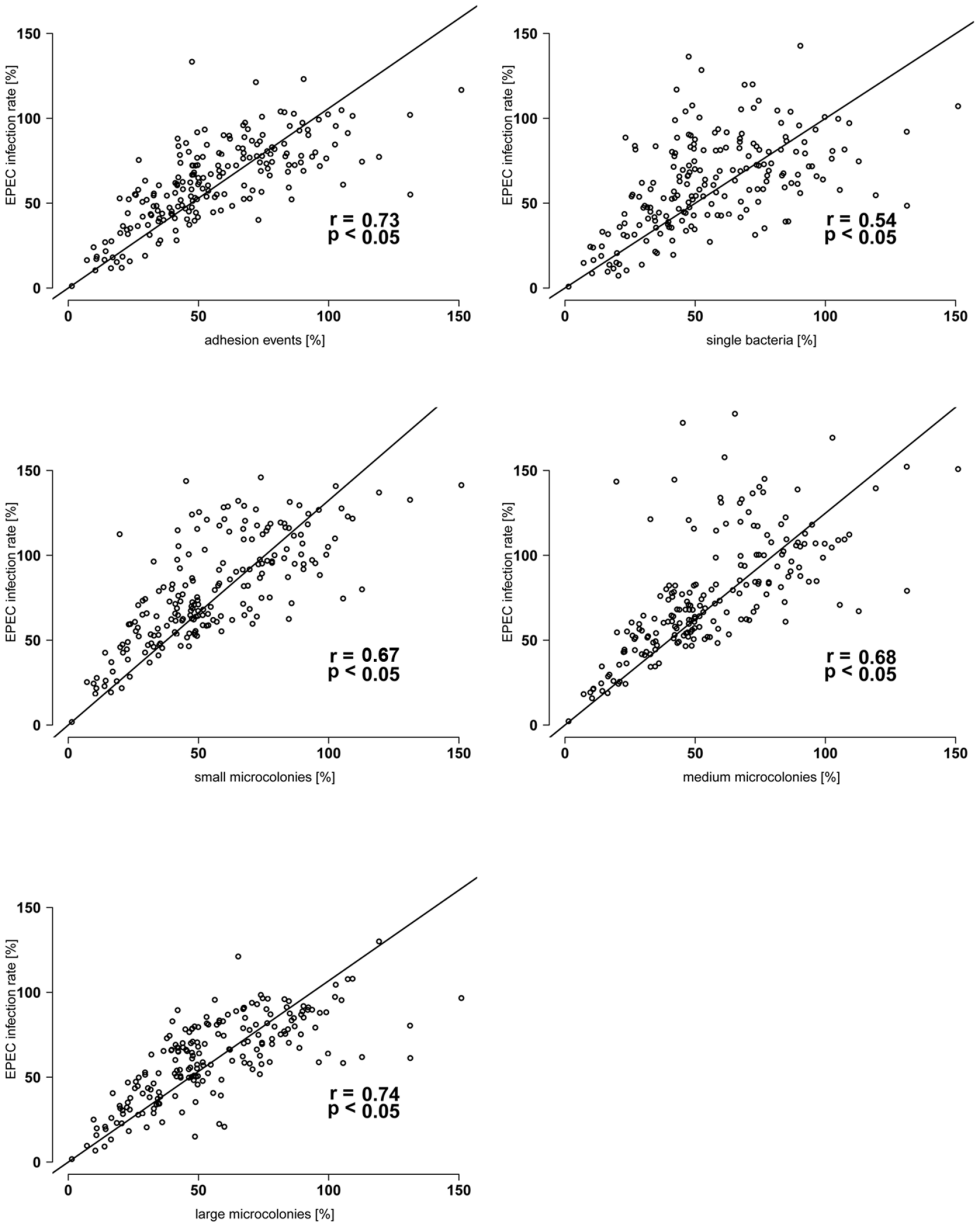
Reduction of EPEC infection rate is associated with the reduction of microcolonies. X-axes: EPEC infection rate is the number of all EPEC bacteria present on IPEC-J2 cells and was determined in an EPEC inhibition assay: *E. coli* (n = 197, domestic pig and wild boar *E. coli* together) were incubated over 2 h with IPEC-J2 cells. Non-adherent bacteria were removed by washing. Subsequently, IPEC-J2 cells were incubated with EPEC. After 6 h non-adherent bacteria were removed by washing. The EPEC infection rate was calculated in relation to EPEC mono-infection (no domestic pig or wild boar *E. coli* pre-incubation = 100%). Y-axes: EPEC adhesion events, single bacteria, small microcolonies, medium size microcolonies and large colonies were calculated in relation to EPEC mono-infection (no domestic pig or wild boar *E. coli* pre-incubation = 100%). Adhesion event: one EPEC formation (including single bacteria as well as microcolonies) which presumably based on one initial adherent EPEC bacterium, single bacterium: one bacterium with no contact to other bacteria, small microcolony: one microcolony consisting of 2–10 bacteria, medium size microcolony: one microcolony consisting of 11–20 bacteria, large microcolony: one microcolony consisting of more than 20 bacteria. If an *E. coli* isolate reduced the EPEC infection rate than preferentially adhesion events and numbers of microcolonies were reduced.

We additionally tested the effect of *E. coli* isolates on EPEC microcolony formation. Inhibition of microcolony formation includes inhibition of EPEC adhesion and growth on epithelial cells. Analysis showed that if *E. coli* reduced the EPEC infection rate then the additional parameters also were reduced (all p<0.05) in the following order: large microcolonies (most reduced, r = 0.74)>medium size microcolonies (r = 0.68)>small microcolonies (r = 0.67)>single bacteria (r = 0.54). Reduction of EPEC infection rate was also highly correlated with reduction of adhesion events, meaning that adhesion and microcolony growth were reduced (Figure 5). If microcolony growth is inhibited, attached bacteria tend to remain as single bacteria, helping to explain a lower correlation between the reduction in EPEC infection rate with the reduction in the occurrence of single bacteria.

Finally, we chose two isolates for further study of their probiotic activity. These tests included both our screening method and conventional cell lysis assays in which epithelial cells with adherent bacteria are lysed, the lysates plated and colony forming units enumerated after overnight incubation [13]. One *sfa/foc*-positive isolate (from a wild boar) and one *sfa/foc*-negative isolate (from a domestic pig) were tested in six independent assays using our fast-screening method. The *sfa/foc*-positive isolate reduced the EPEC infection rate to 12.0±11.6% and the *sfa/foc*-negative isolate reduced the EPEC infection rate to 27.1±17.8%. Using the more labour-intensive cell lysis assay, which was repeated once, the *sfa/foc*-positive isolate reduced the EPEC infection rate to 0.3±0.3% and the *sfa/foc*-negative isolate reduced the EPEC infection rate to 31.6±2.2%, confirming the data obtained using the fast-screening method.

## Discussion

Adhesion of bacteria is a prerequisite for successful colonization of the intestine, which includes defending intestinal microhabitats against competitors, and may be a prerequisite for infection and disease. Previous studies on *E. coli* adhesion and possible probiotic effects against enteropathogens focused on single or low numbers of isolates and genes [1], [2], [12], [14]. Conclusions of such studies are specific for these isolates. To elucidate more globally *E. coli*'s potential as a probiotic and its adhesive abilities, fast-screening methods are needed. We attempted to address this issue by adaptation of an automated fluorescent pattern interpretation system to screen cultured intestinal cells for bacterial adherence. A porcine intestinal epithelial cell line (IPEC-J2) was cultivated in 96-well microtiter plates, and adhesion and probiotic assays were analyzed using fluorescence-labeling of epithelial cells and bacteria. Results were associated with phylogenetic origin and the occurrence of various bacterial genes.

Though *uspA* might be specific to *E. coli* [24], *uspA*-negative *E. coli* isolates were identified. In accordance to other studies about commensal *E. coli* of different host origin, isolates from domestic pigs were predominantly members of phylogenetic group A, while B2 group members occurred at the lowest prevalence. Interestingly, *E. coli* from wild boars were much more likely to be assigned to phylogenetic group B2 [17]. iVAGs were only sporadically detected in *E. coli* from domestic pigs, which is similar to the findings of others [46], [47]. Testing our non-hemolytic isolates for eVAGs our isolates could carry a broad variety, with as many as 22 eVAGs and a median of 8.5 eVAGs. On average, non-hemolytic *E. coli* isolates from domestic pigs carried lower numbers of eVAGs than hemolytic strains but similar numbers to the *E. coli* of wild boar [17], [48]. Conclusively, hemolytic strains are equipped with more virulence factors and are more similar to extraintestinal pathogenic *E. coli* than non-hemolytic isolates.

With the exception of *gimB* and *iha*, all eVAGs were present in our study. Compared to wild boar *E. coli*, several eVAGs were more frequent in *E. coli* from domestic pigs, e.g., *papC* and *iroN,* or less frequent, e.g., *chuA* and *hra*. Such differences should be verified in future studies.

*E. coli* from domestic pigs also carried a broad variety of genes coding for antimicrobial resistance. This fact is recognized and most resistance genes (e.g. *bla*_TEM_, *tet*(A), *tet*(B), *sul1*, *aadA*) were observed also in other domestic pig *E. coli* in one of our previous studies [49]. It remains unknown why they are present in the *E. coli* of pigs in the apparent absence of selection pressure from the respective antimicrobial substance.

Genes coding for resistance against metal ions (mRGs) support bacterial survival in the environment with toxic concentrations of such ions released in the environment either from natural weathering of rocks or through industrial activities [50], [51]. mRGs also support carriage of mobile genetic elements coding for colonization or virulence factors and bacterial persistence [52]–[54]. Data on prevalence of mRGs in *E. coli* populations are not available for human or animal isolates. In our study mRGs coding for resistance against arsenic, copper and silver were frequently present and mRGs coding for resistance against mercury and tellurite were also detectable indicating that such genes are well distributed in intestinal *E. coli* of domestic pigs. The significant higher prevalence of mRGs in *E. coli* from domestic pigs compared to *E. coli* from wild boars indicates that industrial pig production supports occurrence and selection of *E. coli* resistant against metal ions.

More than 90% of our *E. coli* isolates (domestic pig and wild boar isolates together) showed little adherence to IPEC-J2 cells after two-hour incubation with less than 1 bacterium per epithelial cell. Adhesion rates increased during the six-hour incubation period. This can be due to bacterial growth in the supernatant over time. However, not all bacteria showed increased adherence over time and some actually showed a decline, indicating that isolates carried different and, perhaps, unknown adhesins or that they expressed adhesins in the presence of epithelial cells resulting in higher adhesion.

Adhesion rates of isolates did appear to be associated with F1C fimbriae (*sfa*/*foc* gene). F1C fimbria was confirmed as an adhesion factor in other studies [2], [15], [16]. Other adhesins were not definable though many virulence and resistance factors were included as well as defined adhesins like type 1 fimbriae, F1C fimbriae, P fimbriae, one APEC fimbrial adhesion, one fimbria associated with meningitis, flagellae, curli, one APEC autotransporter and others (see Table 1). Since an artificial cell culture model is an imperfect model of the mammalian intestine in life, we cannot definitively state that iVAGs, eVAGs, aRGs and mRGs do not play a role in adhesion to the intestinal epithelium *in vivo*. Since we found isolates, which were highly adherent after 2 h and/or 6 h, but lacked *sfa*/*foc* other undefined adhesins were responsible for high adhesion.

Here, adhesion after 6-hour incubation was higher for *E. coli* from domestic pigs, as compared to *E. coli* from wild boars. This is in contrast to our previous study, where we reported higher adhesion rates of *E. coli* from wild boar after a 3-hour incubation period [55]. Differences between these two studies might be due to different experimental protocols including the difference in incubation times, use of 12- or 96-well cell culture plates or washing with a shaker with 100 rpm vs. with a pipette.

Most of our *E. coli* isolates inhibited infection of intestinal epithelial cells by EPEC, a pathogen that frequently causes diarrhea in animal and human hosts. In weaned pigs EPEC leads to serious acute diarrhea [56], and in humans, EPEC infections particularly affect infants and toddlers and are associated with high mortality rates in developing countries [57]. Thus, this initial finding that several *E. coli* isolates can have a strong probiotic effect against EPEC could have important implications for human and animal health. Also significant is the finding that our screening method has utility in efficiently identifying probiotic candidates.

Interestingly, *E. coli* of domestic pigs inhibited EPEC infection significantly better than *E. coli* of wild boar. This finding might be partially explained by the greater adhesion of *E. coli* from domestic pigs to IPEC-J2 cells since adhesion is important for *E. coli*'s probiotic activity of *E. coli* [2]. However, here probiotic activity was not clearly associated with adhesive ability. Indeed, lowly-adherent bacteria could also inhibit EPEC infection to the same extent as better adhering isolates. If isolates inhibited EPEC infection then predominantly initial adhesion of EPEC to IPEC-J2 cells as well as microcolony formation were affected indicating that different infection steps of this enteropathogen are influenced.

In conclusion, we characterized 104 non-hemolytic *E. coli* isolates from clinically healthy domestic pigs for the occurrence of virulence and resistance genes and adhesion to cultured intestinal cells and compared these results to those from characterization of 93 *E. coli* isolated from wild boar. That *E. coli* from domestic animals carry more aRGs than *E. coli* from wild animals is well recognized and linked to industrial animal production. That this might the case also for mRGs is supported by our new data. Adhesion rates markedly differed between single isolates. Most isolates were lowly adherent to non-adherent. However, some strains were strongly adherent and carried the gene known to encode F1C fimbriae. Other adhesins–responsible for high adhesion and increased adhesion during longer incubation times - have to be identified in future studies. High adhesion was not necessary for a probiotic effect against EPEC. In general, probiotic effects of *E. coli* against EPEC were marked by reduction in initial EPEC adhesion as well as a reduction in later microcolony growth. Finally, our screening method, based on automated fluorescence microscopy employing modern pattern recognition algorithms enabled us to efficiently screen a large number of bacteria for adhesion and probiotic effects, as compared to conventional cell lysis assays.
